# Investigating Patterns for Self-Induced Emotion Recognition from EEG Signals

**DOI:** 10.3390/s18030841

**Published:** 2018-03-12

**Authors:** Ning Zhuang, Ying Zeng, Kai Yang, Chi Zhang, Li Tong, Bin Yan

**Affiliations:** 1 China National Digital Switching System Engineering and Technological Research Center, Zhengzhou 450002, China; zhuangningyu12@163.com (N.Z.); yingzeng@uestc.edu.cn (Y.Z.); ykfer09@163.com (K.Y.); zcboluo@163.com (C.Z.); tttocean@163.com (L.T.); 2 Key Laboratory for NeuroInformation of Ministry of Education, School of Life Science and Technology, University of Electronic Science and Technology of China, Chengdu 611731, China

**Keywords:** self-induced emotion recognition, electroencephalogram (EEG), features, electrodes, neural patterns

## Abstract

Most current approaches to emotion recognition are based on neural signals elicited by affective materials such as images, sounds and videos. However, the application of neural patterns in the recognition of self-induced emotions remains uninvestigated. In this study we inferred the patterns and neural signatures of self-induced emotions from electroencephalogram (EEG) signals. The EEG signals of 30 participants were recorded while they watched 18 Chinese movie clips which were intended to elicit six discrete emotions, including joy, neutrality, sadness, disgust, anger and fear. After watching each movie clip the participants were asked to self-induce emotions by recalling a specific scene from each movie. We analyzed the important features, electrode distribution and average neural patterns of different self-induced emotions. Results demonstrated that features related to high-frequency rhythm of EEG signals from electrodes distributed in the bilateral temporal, prefrontal and occipital lobes have outstanding performance in the discrimination of emotions. Moreover, the six discrete categories of self-induced emotion exhibit specific neural patterns and brain topography distributions. We achieved an average accuracy of 87.36% in the discrimination of positive from negative self-induced emotions and 54.52% in the classification of emotions into six discrete categories. Our research will help promote the development of comprehensive endogenous emotion recognition methods.

## 1. Introduction

Given that emotion plays an important role in our daily lives and work, the real-time assessment and regulation of emotions can improve our lives. For example, emotion recognition will facilitate the natural advancement of human–machine interactions and communication. Furthermore, recognizing the real emotional state of patients, particularly those of patients with expression problems, will help improve the quality of medical care. In recent years, emotion recognition based on EEG signals has gained considerable attention. The method of emotion recognition is a crucial factor in human-computer interaction (HCI) systems, which will effectively improve communication between humans and machines [1,2].

However, emotion recognition based on EEG signals is challenging given the vague boundaries and individual variations presented by emotions. Moreover, in theory, we cannot obtain the “ground truth” of human emotions, that is, the true label of EEG that correspond to different emotional states, because emotion is a function of time, context, space, language, culture, and races. Therefore, researchers have used various affective materials, such as images, sounds, and videos, to elicit emotions. Affective video materials are widely used by researchers given that these materials can expose subjects to real-life scenarios through the visual and aural stimuli that they provide.

DEAP is a multimodal dataset used to analyze human affective states. This dataset contains EEG and peripheral physiological signals acquired from 32 participants as they watched 40 one-minute-long excerpts of music videos [3]. MAHNOB-HCI is another multimodal database of recorded responses to affective movie stimuli. A multimodal setup was established for the synchronized recording of face videos, audio signals, eye-gaze data, and peripheral/central nervous system physiological signals of 27 participants [4]. Zheng et al. developed a SEED dataset to investigate stable patterns over time for emotion recognition from EEG. Fifteen subjects participated in the experiment, and each subject was required to perform the experiment for three sessions. The time interval between two sessions is one week or longer [5]. Liu et al. constructed a standard database of 16 emotional film clips selected from over one thousand film excerpts and proposed a system for real-time recognition of movie-induced emotion through the analysis of EEG signals [6].

Various features and extraction methods based on the above datasets have been proposed for the recognition of emotions from EEG signals. These methods include time domain, frequency domain, joint time-frequency analysis, and empirical mode decomposition (EMD) techniques [7]. 

The statistical parameters of EEG series, including first and second difference, mean value, and power, are usually utilized as features in time domain techniques [8]. Nonlinear features, including fractal dimension [9,10], sample entropy [11] and nonstationary index [12] have been utilized for emotion recognition. Hjorth features [13], and higher order crossing features [14] had also been used in EEG studies [15,16].

Time-frequency analysis is based on the spectrum of EEG signals, and the energy, power, power spectral density and differential entropy (DE) [17] of a certain subband are utilized as features. Short-time Fourier transform (STFT) [18,19], Hilbert-Huang transform [20,21] and discrete wavelet transform [22,23,24,25] are the most commonly used techniques for spectral calculation. Higher frequency subbands, such as Beta (16–32 Hz) and Gamma (32–64 Hz) bands, have been verified to outperform lower subbands in emotion recognition [3,26]. 

Mert et al. extracted entropy, power, power spectral density, correlation, and asymmetry of intrinsic mode functions (IMF) as features through EMD and then utilized independent component analysis (ICA) to reduce the dimensions of the feature set. Classification accuracy of emotions was computed with all the subjects merged together [27]. Zhuang et al. utilized the multidimensional information of IMF, the first difference of time series, the first difference of phase, and normalized energy as features. They then verified the classification performance of their method with the DEAP dataset and found that the classification accuracy is superior to DE of the Gamma band [28].

Other features extracted from electrode combinations, such as the coherence and asymmetry of electrodes in different brain regions [29,30,31], and graph-theoretic features [32], have been utilized. Jenke et al. compared the performance of different features and obtained a guiding rule for feature extraction and selection [33].

Some other strategies, such as the utilization of deep networks, have also been investigated to improve classification performance. Zheng used a deep neural network to investigate critical frequency bands and channels for emotion recognition [34]. Yang used a hierarchical network with subnetwork nodes for emotion recognition [35]. Li et al. designed a hybrid deep-learning model that combines the convolutional neural network and recurrent neural network to extract task-related features. They then performed experiments with the DEAP dataset [36]. 

All datasets and methods for emotion recognition are based on external affective stimuli. However, few studies on self-induced emotion recognition from EEG have been conducted despite their importance to endogenous emotion recognition. Liu et al. investigated the profile of autonomic nervous responses during the experience of five basic self-induced emotions: sadness, happiness, fear, anger, surprise and neutral. ECG and respiratory activity of fourteen healthy volunteers were recorded during their reading passages with five basic emotional tones and neutral tone to elicit corresponding endogenous emotions. They found that it was feasible and effective to recognize users’ affective states based on peripheral physiological response patterns of ECG and respiratory activities. However, their research did not include the patterns of EEG signals for self-induced emotion [37]. The stability, performance and neural patterns of self-induced emotion recognition based on EEG signals remain unknown. Moreover, whether self-induced emotion and affective stimuli-induced emotion share commonalities remains a point of contention. The main contributions of this study to the EEG-based emotion recognition can be summarized as follows:

(1) We have developed an emotional EEG dataset for the evaluation of stable patterns of self-induced emotion across subjects. To the best of our knowledge, a public EEG dataset for analyzing the classification performance of stable neural patterns in the recognition of self-induced emotion is unavailable.
(2) We systematically compared self-induced emotion with movie-induced emotion and found that these two types of emotions share numerous commonalities.
(3) We analyzed the important features, electrode distribution, and average neural patterns of different self-induced emotions. Our analytical results will support future efforts for real-time recognition of endogenous emotions in real life.
(4) We confirmed that self-induced emotions exhibit subject-independent neural signatures and relatively stable EEG patterns at critical frequency bands and brain regions.

This paper is structured as follows: a detailed description of the experimental setup is presented in Section 2. A discussion of the methodology is provided in Section 3. The classification results and analysis are presented in Section 4. The discussion is given in Section 5, and the conclusion is given in Section 6.

## 2. Experiment Setup

We designed a novel emotion experiment to collect EEG data for the investigation of different emotional states. Our experiment is different from other existing publically available datasets. 

### 2.1. Experimental Protocol

We designed an experiment and recorded the EEG signals of 30 participants. Each participant watched 18 Chinese movie clips from the Chinese affective video system [38]. These movie clips were intended to elicit six discrete emotions, including joy, neutrality, sadness, disgust, anger, and fear. 

**Figure 1:**
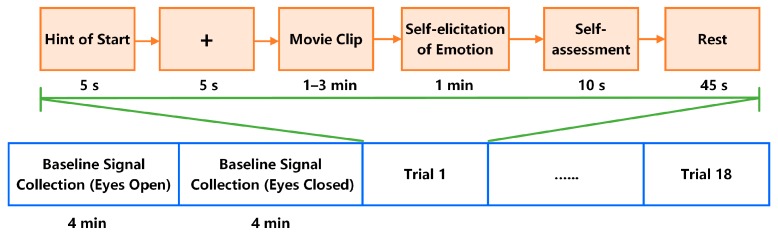
Experimental protocol.

These emotional movie clips contained scenes and audio that exposed participants to real-life scenarios and elicited strong subjective and physiological changes. The details of the movie clips used in our experiment are listed in Table 1. All six categories of movie clips were randomly presented to the participants. The participants performed a practice trial to familiarize themselves with the system. A short video was shown during the unrecorded trial. Next, the researcher started the EEG signal recording and left the room. The participant then began the experiment by pressing a key on the keyboard. The formal experiment started with 4 min of baseline recording, during which a fixation cross was displayed to the participant. Next, the participants were asked to close their eyes and stay relaxed. During this period, a 4 min baseline signal was recorded while the participant kept their eyes closed. Then, the 18 movie clips were presented in 18 trials, each consisting of the following steps (see Figure 1):

(1) 5 s display of the current trial number to inform the participants of their progress.
(2) 5 s of baseline signal collection (fixation cross).
(3) Display of the movie clips.
(4) 1 min of self-elicitation of emotion, during which participants closed their eyes and attempted to recall scenes from the movie clip that they had just watched.
(5) 10 s of self-assessment for arousal and valence.
(6) 45 s of rest time.

**Figure 2:**
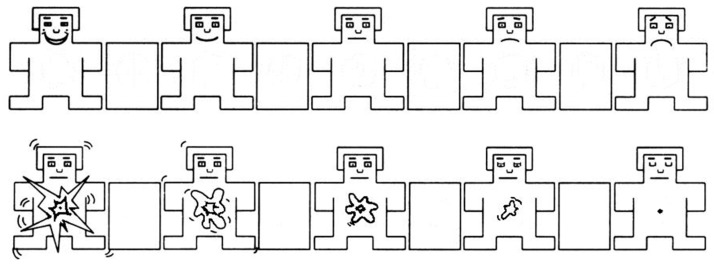
Self-assessment for arousal and valence.

After watching each movie clip, we asked the participants to recall a scene from the movie to self-elicit emotion. This method enabled us to tag self-induced emotion and movie-induced emotion in one experiment. Then, the participants opened their eyes and self-assessed their levels of arousal and valence. Self-assessment manikins [39] were used to visually represent arousal and valence levels (see Figure 2). The manikins were displayed in the middle of the screen with the numbers 1–9 printed below them. Participants used a keyboard to directly input the number that corresponded to their arousal and valence levels. At the end of each trial, participants had 45 s of rest. They could drink water and relax.

### 2.2. EEG Data Acquisition

**Figure 3:**
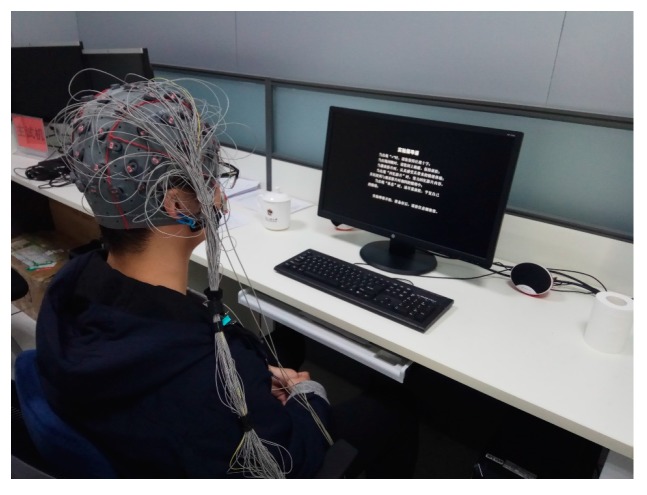
Experimental environment.

Participants were selected through interviews and questionnaire administration. Beck Anxiety Inventory [40], Hamilton Anxiety Rating Scale [41], and Hamilton Rating Scale for Depression [42] tests were administered to exclude individuals with anxiety, depression, or physical abnormalities, as well as those using sedatives and psychotropic drugs. Finally, 30 native Chinese undergraduate and graduate students (20 males and 10 females) with an average age of 23.73 years (range = 18–35, SD = 2.98) participated in our experiment. All participants were right-handed with normal or corrected-to-normal vision and normal hearing. Before the experiments, the participants were informed about the experiment and were instructed to sit comfortably, watch the forthcoming movie clips attentively without diverting their attention from the screen, and refrain as much as possible from overt movements. The movie clips were presented on a 23-inch screen (refresh frequency = 60 Hz). To minimize eye movements, all stimuli were displayed on the center of the screen. A stereo amplifier was used, and the volume was set at a suitable level. The software E-Prime 2.0 (Psychology Software Tools, Sharpsburg, GA, USA) was used to present stimuli, mark synchronization labels, and record the participants’ ratings. Figure 3 shows the moment before the start of the experiment.

**Figure 4:**
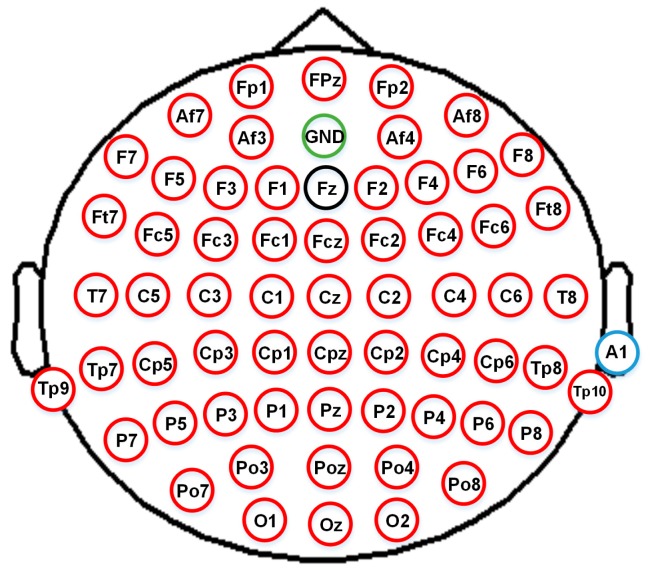
EEG cap layout for 62 channels.

EEG signals were recorded with a g.HIamp System (g.tec Medical Engineering, Linz, Austria). The parameters of the recording system were set in accordance with Table 2. The layout of 62 electrodes followed the international 10–20 system, as shown in Figure 4. The Fz electrode was used for reference calculation. Thus, the number of effective electrodes was 61. 

## 3. Method

**Figure 5:**
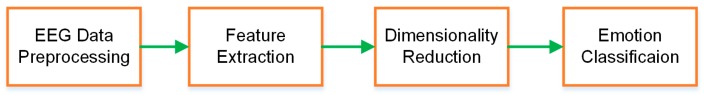
Block diagram of emotion recognition.

EEG signals were preprocessed to remove eye artifacts. Then, two types of features were extracted: DE based on STFT and the first difference of IMF based on EMD. Then, the minimal redundancy–maximal relevance (MRMR) algorithm was utilized for feature dimension reduction. Finally, the retained features were fed into support vector machine (SVM) for classification. The whole process is shown in Figure 5.

### 3.1. Data Preprocessing

**Figure 6:**
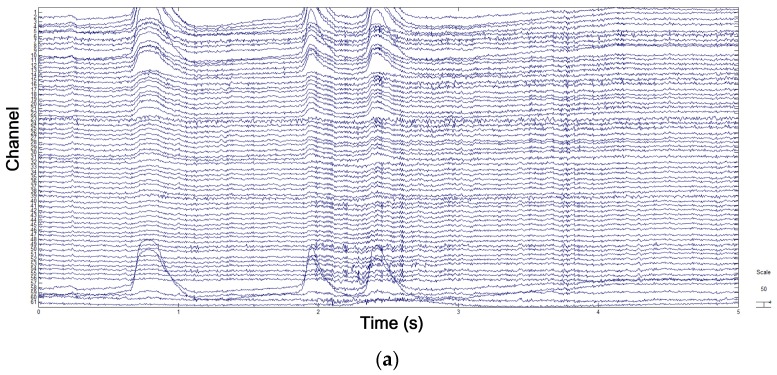

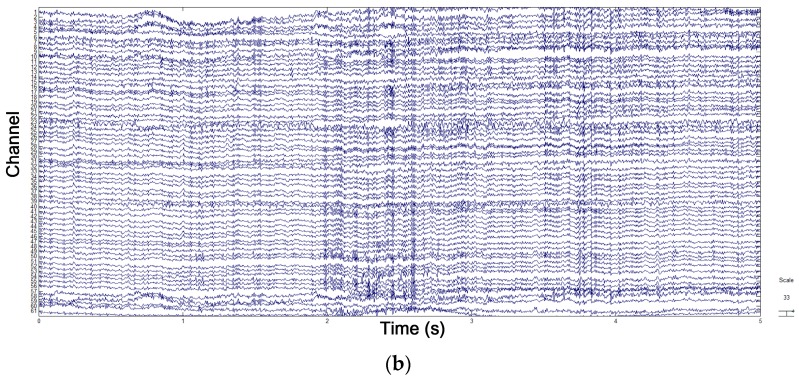
EEG signals before and after the removal of EOG artifacts. (**a**) EEG signals contaminated by EOG artifacts. (**b**) EEG signals without EOG artifacts.

To ensure that all emotional EEG data had the same length, we took the last 50 s of each video and of the 1 min self-elicitation of emotion for analysis. Before feature extraction, high-frequency interferences were filtered out from EEG signals by using a band-pass filter with a range of 0.1–80 Hz. Then, electrooculography (EOG) artifacts were removed by the blind-source analysis algorithm FastICA [43]. Each subject’s signal was decomposed into 61 independent components (ICs). Then, EOG artifacts were selected and removed. Figure 6a,b illustrate EEG data before and after the removal of EOG artifacts. Finally, the 5 s of pretrial baseline was removed from the EEG signals.

### 3.2. Feature Extraction

**Figure 7:**
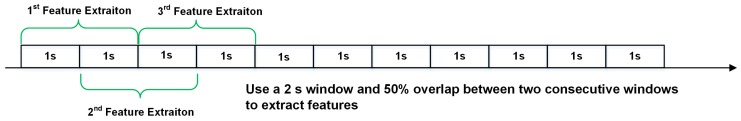
Feature extraction process.

In this study, we segmented EEG signals by using a 2 s window and a 50% overlap between two consecutive windows. Figure 7 shows the feature extraction process. Each emotional EEG data lasted for 50 s, and 2 s of EEG signals were extracted as samples. Therefore, we acquired 882 labeled samples for each subject who watched 18 movie clips. Two types of features were utilized for emotion recognition: DE based on STFT and the first difference of component IMF1 based on EMD.

#### 3.2.1. DE Based on STFT

We utilized STFT for the time-frequency analysis of EEG signals. The window length of STFT was 128 with 50% overlap. The original EEG signal is *s*(*t*). After STFT, we acquired:

(1) $${\mathrm{STFT}}_{s,\gamma }(t,f)=\int_{-\infty }^{+\infty }s\left(\tau \right)\gamma^{*}\left(t-\tau \right)e^{-j2\pi f\tau }d\tau =\int_{-\infty }^{+\infty }s\left(\tau \right)\gamma^{*}{}_{t,f}e^{-j2\pi f\tau }$$

From STFT*_s,γ_*(*t,f*), we calculated the power of *δ*, *θ*, *α*, *β*, and *γ* bands in accordance with Table 3, as follows:

(2) $$spectrogram\left\{s[n]\right\}\left(m,f_{k}\right)={\left|S\left(m,f_{k}\right)\right|}^{2}$$

DE is defined as follows:

(3) $$\mathrm{DE}=\log\left({\left|S\left(m,f_{k}\right)\right|}^{2}\right)$$

Given that the effective electrode of EEG signals is 61 channels, we extracted 305 DE features from each sample.

#### 3.2.2. First Difference of IMF Based on EMD

EMD decomposes EEG signals into a set of IMFs through an automatic shifting process. Each IMF represents the different frequency components of original signals and should satisfy two conditions: (1) For the whole dataset, the number of extreme points and the number of zero crossings must be either equal or differ at most by one. (2) At each point, the mean value calculated from the upper and lower envelope must be zero [7].

**Figure 8:**
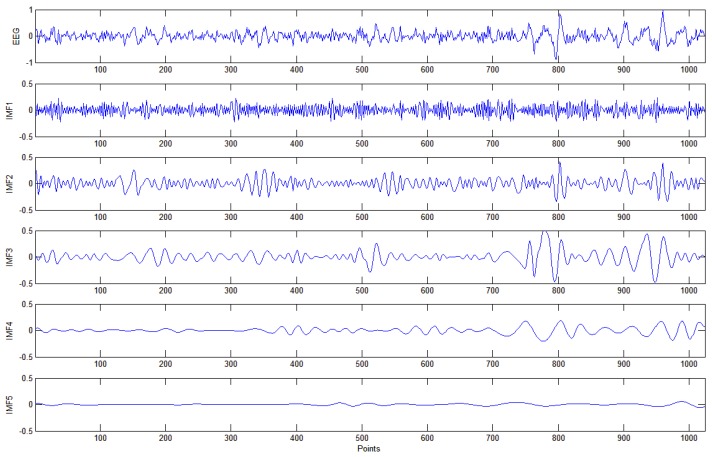
EEG signals and their corresponding first five IMFs.

EMD functions similarly to an adaptive high-pass filter. It first shifts out the fastest changing component and smoothens the oscillation of IMF as the level of IMF increases. Each component is band-limited and reflect the characteristics of instantaneous frequency. Figure 8 shows a segment of original EEG signals and the corresponding first five decomposed IMFs.

As stated in [28], the components of IMF1 with high oscillation frequency play a more important role in emotion recognition than those with low oscillation frequency. Therefore, we extracted the first difference *D_t_* of time series IMF1 as a feature. For an IMF1 component with *N* points, *IMF*{*imf*_1_, *imf*_2_, …, *imf_N_*}, *D_t_* is defined as follows:

(4) $$D_{t}=\frac{1}{N-1}\sum_{n=1}^{N-1}\left|imf(n+1)-imf(n)\right|$$

We utilized log(*D_t_*) as a feature. The effective electrode of EEG signals has 61 channels. Thus, for each sample, we extracted 366 features, 305 DE features, and 61 features on the basis of the EMD strategy.

### 3.3. Dimensionality Reduction

Previous research [33] has shown that the MRMR algorithm developed by Ding and Peng [44] is suitable for emotional feature selection and outperforms other methods, such as ReliefF [45] and effect-size-based feature selection methods. MRMR utilizes mutual information to characterize the suitability of a feature subset. Mutual information between two random variables *x* and *y* is defined as:

(5) $$I\left(x;y\right)=\iint p\left(x,y\right)\log\frac{p\left(x,y\right)}{p\left(x\right)p\left(y\right)}dxdy$$

where *p*(*x*) and *p*(*y*) are the marginal probability density functions of *x* and *y*, respectively, and *p*(*x,y*) is their joint probability distribution. If *I*(*x*;*y*) equals zero, the two random variables *x* and *y* are statistically independent.

The MRMR method aims to optimize two criteria simultaneously: (1) Maximal-relevance criterion *D*, which aims to maximize average mutual information *I*(*x_i_*;*y*) between each feature *x_i_* and the target vector *y*. (2) Minimal redundancy criterion R, which aims to minimize the average mutual information *I*(*x_i_*;*y*) between two features. The algorithm finds near-optimal features by using forward selection. Given an already chosen set *S_k_* of *k* features, the next feature is selected by maximizing the combined criterion *D* − *R*:

(6) $$\underset{x_{j}\in \chi -S_{k}}{\max}\left[I\left(x_{j};y\right)-\frac{1}{k}\sum_{x_{i}\in S_{k}}I\left(x_{j};x_{i}\right)\right]$$

### 3.4. Classification

The extracted features were fed into SVM for classification. SVM is widely used for emotion recognition [46,47] and has promising applications in many fields. In our study, LIBSVM was implemented for SVM classifier with linear kernel function and default parameter setting [48].

## 4. Results

### 4.1. Classification of Self-Induced Emotions

We explored the classification of self-induced emotions by performing three subject-dependent experiments:

● Movie-Induced Emotion Recognition

Movie-induced emotional data were used as the training and testing sets for this classification task. Each subject watched 18 movie clips. In binary classification, samples from joy movie clips (three clips) were classified as positive, and samples from sad, disgust, anger, and fear movie clips (12 clips) were classified as negative. We utilized 49 samples from one movie clip as the testing set and all the other 686 samples from 14 movie clips as the training set to avoid correlations between the training and testing sets. The final accuracy for each subject could be acquired by averaging 15 results from the 15 tested movie clips. To classify emotions into six discrete categories, we utilized 49 × 2 samples from two movie clips of each emotional category as the training set and 49 samples from the one remaining movie clip as the testing set to avoid correlations between the training and testing sets. The final accuracy of each subject could be acquired by averaging three results from the three tested movie clips.

● Self-Induced Emotion Recognition

Self-induced emotional data were used as the training and testing sets for this classification task. Each subject recalled 18 movie clips. In binary classification, samples from the recollection of joy movie clips (three clips) were classified as positive, whereas those from the recollection of sad, disgust, anger, and fear movie clips (12 clips in total) were classified as negative. Each time, we utilized 49 samples from one movie clip as the testing set and all other 686 samples from 14 movie clips as the training set to avoid correlations between the training and testing sets. The final accuracy for each subject could be acquired by averaging 15 results from the 15 test sets. To classify emotions into six discrete categories, we utilized 49 × 2 samples from the recollection of 2 movie clips from each emotion category as a training set and 49 samples from the remaining movie clip as a testing set to avoid correlations between the training and testing sets. The final accuracy for each subject could be acquired by averaging three results from the three test sets.

● Prediction of Self-Induced Emotion through Movie-Induced Emotion

We utilized all 49 × 18 samples from 18 movie-induced emotional data as the training set to establish a classification model. By utilizing the established model, we predicted the categories of self-induced emotion, all 49 × 18 samples from self-induced emotional data as the testing set. 

#### 4.1.1. Classification of Positive and Negative Emotions

Table 4 shows the accuracies of binary classification for 30 participants in three experiment tasks above. The average accuracy for the binary classification of self-induced emotion is 87.36%, which is close to that of movie-induced emotion (87.20%). The average accuracy obtained for the third experiment task is 78.53%, which is far above the random probability of 50%. These findings indicated that self-induced emotion and movie-induced emotion share numerous commonalities. In the future, we can use a model established on the basis of affective-stimulus -induced emotion to predict comprehensively endogenous emotion. 

**Figure 9:**
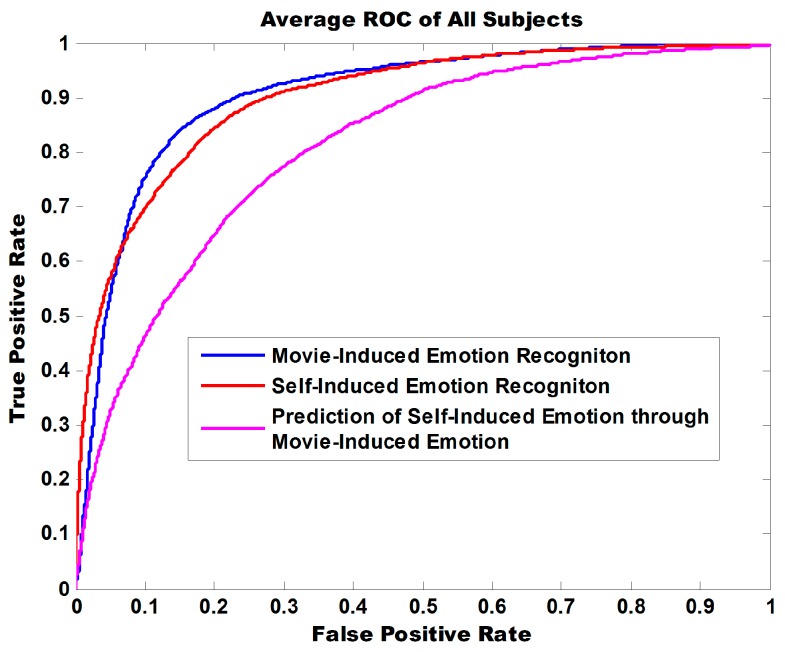
ROC curve of binary emotional classification in three experiment tasks. Positive emotions are distinguished from negative emotions. The AUC values of binary classification for movie-induced emotion recognition, self-induced emotion recognition, and prediction of self-induced emotion through movie-induced emotion are 0.9047, 0.8996, and 0.8102, respectively.

We provided some other strategies for measuring classification performance in the case of an unbalanced training set of positive and negative samples. Figure 9 illustrates the ROC curve of three experiment tasks. The areas under curve (AUC) are 0.9047, 0.8996, and 0.8102 and indicate that the model exhibits robust classification performance in discriminating positive from negative for both self-induced emotions and movie-induced emotions. 

Table 5 provides the F1 score and classification accuracy for binary emotion recognition. The F1 score of positive samples is lower than that of negative samples because the training set contains higher numbers of negative samples than positive samples. The F1 scores of negative samples for three experiment tasks are 0.94, 0.92, and 0.86. The classification performance in F1 score and accuracy for self-induced emotions is similar to that for movie-induced emotions.

#### 4.1.2. Classification of Emotions into Six Discrete Categories

Table 6 shows the accuracies obtained for the classification of the emotions of 30 participants in three experiment tasks. Emotions are classified into six discrete categories. The average classification accuracy for self-induced emotion is 54.52%, which is close to that for movie-induced emotion (55.65%). The average accuracy for the third case, prediction of self-induced emotion through movie-induced emotion, is 49.92%, which is far above the random probability of 16.67%. 

**Figure 10:**
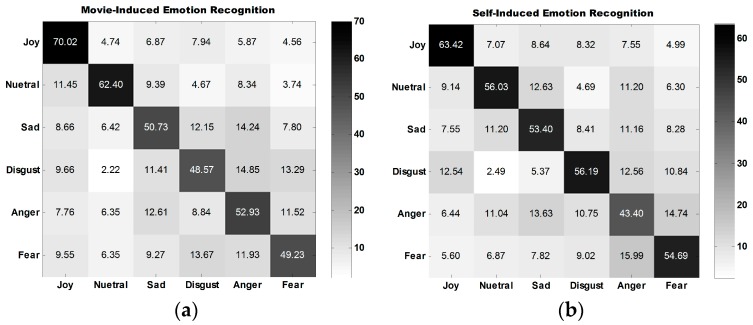

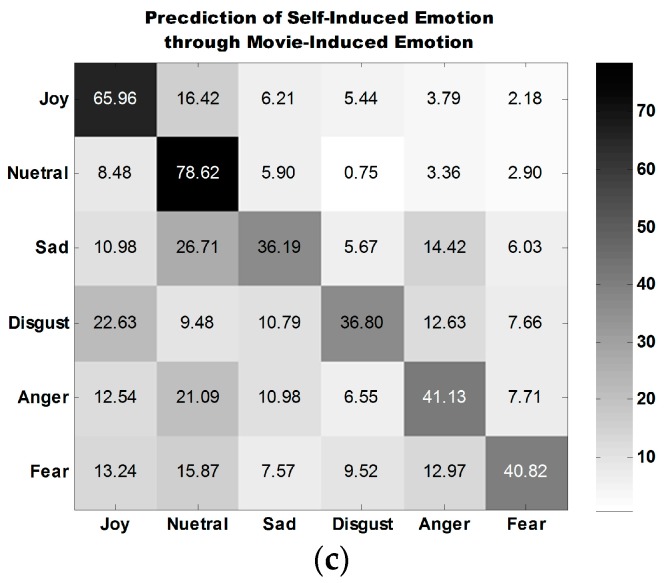
Average confusion matrix for the classification of emotions of 30 participants into six discrete categories. (**a**) Average confusion matrix for movie-induced emotion recognition. (**b**) Average confusion matrix for self-induced emotion recognition. (**c**) Average confusion matrix for prediction of self-induced emotion through movie-induced emotion.

The average confusion matrix of all participants under three experiment tasks is illustrated in Figure 10. Figure 10a,b show that the classification performance for joy is the best, followed by that for neutral emotion.

The classification performance for disgust is the best among the classification performances for all four types of self-induced negative emotions. The classification performance for anger is the best among all classification performances for all four types of movie-induced negative emotions. Figure 10c shows that the model established on the basis of movie-induced emotion exhibits the best prediction performance for self-induced neutral emotion and then for joy. Among the classification performances for four types of negative emotions, that for anger is the best. The four negative emotions are easily misclassified, indicating that negative emotions share some commonalities.

### 4.2. Dimensionality Reduction

For each sample, we extracted 366 features in total. Are these features effective in emotion recognition? Which features and electrodes are more important in self-induced emotion recognition? In this subsection, we utilized MRMR method to analyze the important features, electrodes for self-induced emotion recognition.

**Figure 11:**
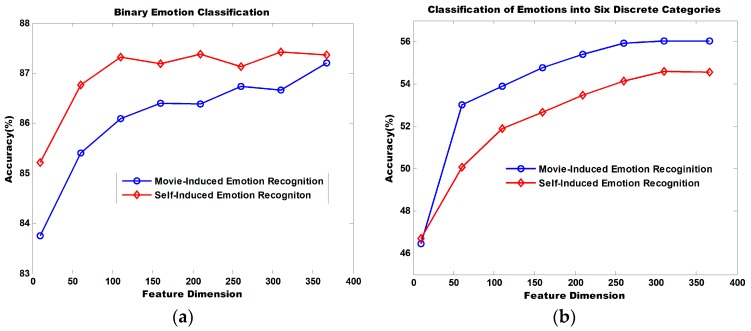
Dimensionality reduction using MRMR. MRMR is used to sort 366 features for each participant. The top 10, 60, 110, 160, 210, 260, and 310 features and all 366 features are utilized for emotion recognition. Average accuracy is computed for all participants. (**a**) Binary emotional classification with different numbers of features. (**b**) Classification of emotions into six discrete categories with different numbers of features.

Figure 11 illustrates the dimensionality reduction performance of the MRMR algorithm. The binary classification of self-induced emotion recognition achieves an accuracy of 85.21% and that of movie-induced emotion achieves an accuracy of 83.75% when the top 10 ranked features sorted by MRMR are selected for recognition. Accuracy increases continuously with the increasing number of utilized features. When 366 features are utilized, the classification accuracy for self-induced emotions is 87.36% and that for movie-induced emotion is 87.20%.

When the top 10 ranked features sorted by MRMR are selected for the classification of emotions into six discrete categories, the classification accuracy for self-induced emotion is 46.70% and that for movie-induced emotion is 46.47%. Accuracy increases continuously as the number of utilized features increases. When 366 features are utilized, the classification accuracy for self-induced emotions is 54.52% and that for movie-induced emotion is 55.65%.

To classify emotions into six discrete categories, we selected the corresponding top 20 electrode distributions in accordance with the ranking of the 366 features sorted by MRMR. The results are shown in Table 7. The DE of electrode TP8 in the Beta band; the DE of electrodes AF7, AF8, FP1, FP2, F6, F8, FC6, FT8, T7, T8, TP8, TP9, TP10, CP6, P8, O1, O2, and Oz of the Gamma band; and the first difference of IMF1 of electrodes T7, T8, and C6 decomposed through EMD play an important role in the classification of movie-induced emotions.

The DE of electrodes AF7, AF8, FP1, FC5, FC6, FT7, FT8, T7, T8, TP7, TP8, TP9, TP10, C5, C6, CP6, P8, O1, and Oz in the Gamma band and the first difference of IMF1 of electrodes FT8, T8, TP10, and CP6 decomposed through EMD play an important role in the classification of self-induced emotions. 

The features of high-frequency bands provide outstanding classification performance. These features include the DE of the Gamma band and the first difference of wave IMF1 with the highest oscillation frequency decomposed through EMD. 

**Figure 12:**
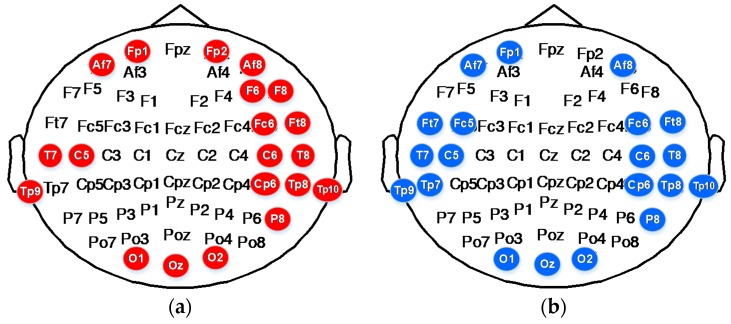
Distribution of top 20 subject-independent features selected on the basis of MRMR ranking. (**a**) Movie-induced emotion recognition. (**b**) Self-induced emotion recognition.

Figure 12 shows the distribution of the top 20 subject-independent electrodes selected on the basis of MRMR ranking. As can be seen from the figure, electrodes C5, C6, CP6, T7, T8, TP8, TP9, and TP10 on the temporal lobe; electrodes AF7, AF8, and FP1 on the prefrontal lobe; and electrodes O1, O2, and Oz on the occipital lobe play important roles in emotion recognition. This finding shows that the neural modes of external movie-induced emotion and internal self-induced emotion share common characteristics. We can use some of the important characteristics of self-induced emotion to lay the foundation for endogenous emotion recognition. 

### 4.3. Neural Signatures and Patterns of Self-Induced Emotion

**Figure 13:**
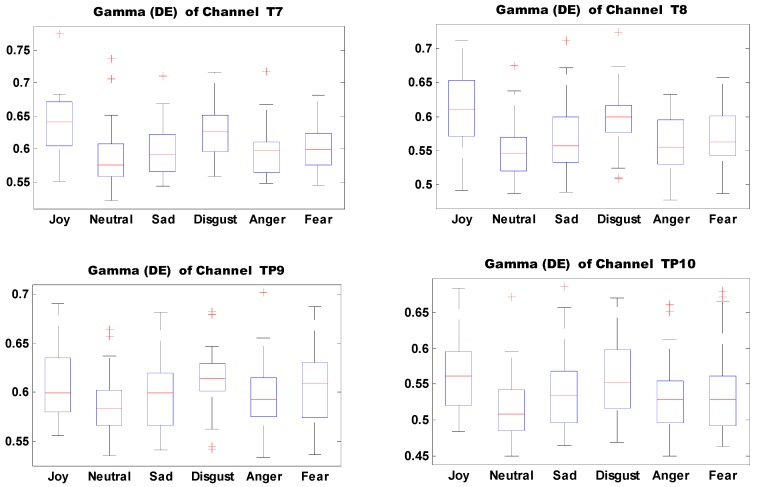

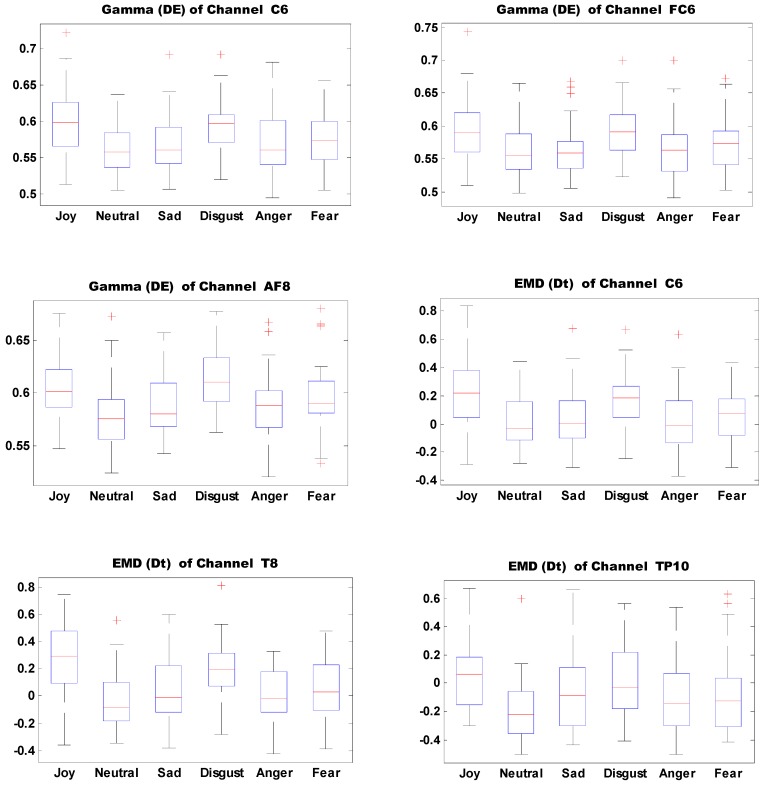
Distribution of 10 important electrode features associated with self-induced emotion.

We analyzed the important features and average neural patterns of different self-induced emotions. Figure 13 shows the boxplots of 10 important features of self-induced emotion. The figure shows that different emotions can be effectively identified by setting proper thresholds for different electrodes and features. For example, joy can be effectively distinguished from sadness, disgust, anger, and fear when the DE threshold of electrode T7 in the Gamma band is set to 0.6.

**Figure 14:**
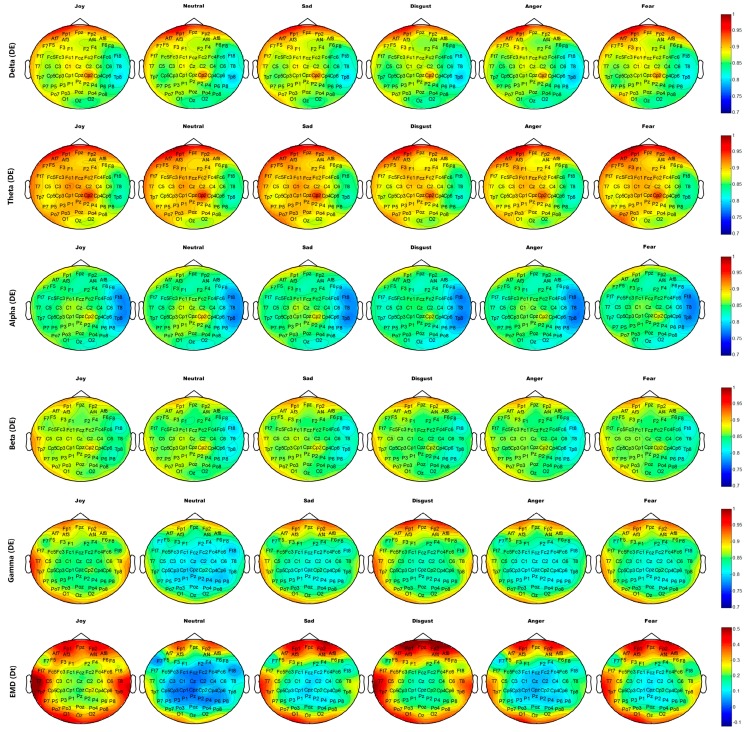
Average neural patterns of different movie-induced emotions in all participants. The DE of frequency band Delta (1–4Hz), Theta (4–8 Hz), Alpha (8–12 Hz), Beta (12–30 Hz), and Gamma (30–64 Hz), and the first difference *D_t_* of IMF1 decomposed through EMD are illustrated from top to bottom.

**Figure 15:**
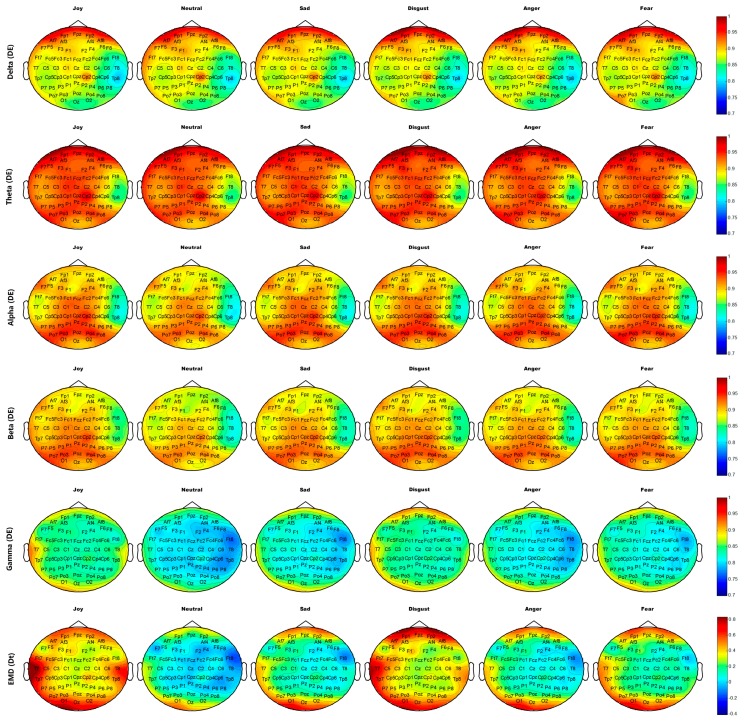
Average neural patterns for different self-induced emotions of all participants. DE of frequency band Delta (1–4Hz), Theta (4–8 Hz), Alpha (8–12 Hz), Beta (12–30 Hz), and Gamma (30–64 Hz) and the first difference *D_t_* of IMF1 decomposed by EMD are illustrated from top to bottom.

Figure 14 and Figure 15 show the average brain topographies of movie-induced emotion and self-induced emotion, respectively. The six discrete emotion categories do not have significantly different brain topographies under the features of DEs of Delta (1–4 Hz), Theta (4–8 Hz), and Alpha (8–12 Hz) band. However, a slight difference in the left temporal lobe is noted under the DE of the Beta (12–30 Hz) band.

Under the DE of the Gamma (30–64 Hz) band, the six discrete categories of self-induced emotion result in significant differences in electrodes T7, T8, TP7, TP8, TP9, and TP10 on both temporal lobes; electrodes O1, O2, and Oz on the occipital lobe; and electrodes AF7, AF8, FP1, and FP2 on the prefrontal lobe. The feature values of both sides of the temporal and occipital lobes for joy are higher than those of other emotions. The feature value of the frontal lobe for disgust is the highest for all emotions. Neutrality has the lowest feature value over the entire brain topography, compared with other five discrete emotion categories. Similar results are observed for movie-induced emotions.

Under feature *D_t_* based on EMD, the six discrete categories of self-induced emotion result in significant differences in electrodes T7, T8, TP7, TP8, TP9, and TP10 on both temporal lobes and in electrodes FPz, FP1, and FP2 on the prefrontal lobe. Disgust has the highest feature value at the prefrontal lobe, and joy has the highest feature value in the left temporal and occipital lobes. Similar results are observed for movie-induced emotions. 

The important electrodes and features inferred from average brain topography are consistent with those selected by MRMR (refer to Section 4.2). Therefore, the neural patterns of self-induced emotion do exist and they have much in common with stimuli-induced emotion. This finding makes sense for real-time recognition of comprehensive endogenous emotion.

## 5. Discussion

Emotion recognition from EEG signals has achieved significant progress in recent years. Previous researches have mainly focused on emotion induced by external affective stimuli, and few studies on the classification of self-induced emotion from EEG are available. The main contributions of this study can be summarized as follows: 

First, we designed an experiment that considers two types of emotions: movie-induced emotion and self-induced emotion. Thirty participants took part in our experiment, and we developed an EEG-based dataset for the evaluation of the patterns of self-induced emotion across subjects.

Second, we evaluated classification performance for self-induced emotions. We achieved an average accuracy of 87.36% in discriminating positive from negative emotions and an average accuracy of 54.52% in classifying emotions into six discrete categories. We achieved similar accuracies for classifying movie-induced emotions. We also utilized movie-induced emotional data as a training set to establish a classification model. We used this model to classify self-induced emotions and achieved 78.53% accuracy in discriminating positive from negative emotions and 49.92% accuracy in classifying emotions into six discrete categories. 

Third, we analyzed the important features and distributions of electrodes through MRMR algorithm. We found that the DE of the Gamma band and the first difference of IMF1 decomposed through EMD have good classification performance. These important electrodes are distributed in the bilateral temporal lobe (C5, C6, CP6, T7, T8, TP8, TP9, and TP10), the prefrontal lobe (AF7, AF8, and FP1), and the occipital lobe (O1, O2, and Oz). We also discovered that self-induced emotion and movie-induced emotion share numerous commonalities.

Finally, by analyzing the average brain topography of all the participants over all experimental sessions, we obtained the neural patterns of self-induced emotion as follows: Disgust is associated with the highest feature value of the prefrontal lobe; joy is associated with high feature values of bilateral temporal lobe and occipital lobes; and negative emotions elicit asymmetries in the bilateral temporal lobe. Moreover, the important brain regions and electrodes that we identified on the basis of average brain topography are consistent with those selected through the MRMR algorithm. 

Our study is limited by our small sample size. We only collected EEG signals from 30 participants. In the future, we will collect additional EEG signals to verify our analysis and conclusion. In addition, we will investigate the real-time recognition of comprehensive endogenous emotion to promote the practical application of emotion recognition based on EEG signals.

## 6. Conclusions

We compiled a dataset comprising the EEG signals of 30 participants for the analysis of self-induced emotion. Then, we identified EEG features, electrode distribution and stable neural patterns that are significantly associated with self-induced emotion. We found that the DE of the Gamma band and the first difference of IMF1 decomposed through EMD have better performances in emotion recognition than other features. The roles of electrodes distributed in the bilateral temporal (C5, C6, CP6, T7, T8, TP8, TP9, and TP10), prefrontal (AF7, AF8, and FP1), and occipital (O1, O2, and Oz) lobes are more important in the discrimination of self-induced emotion than those of electrodes distributed in different regions. In addition, self-induced emotions provide characteristic neural patterns. For example, disgust is associated with the highest feature values in the prefrontal lobe; joy is associated with high feature values in the bilateral temporal and occipital lobes; and negative emotions elicit apparent asymmetries in the bilateral temporal lobe. Moreover, we discovered that self-induced and movie-induced emotions share many commonalities. Our research lays a substantial foundation for real-time recognition of comprehensive endogenous emotion. For the future work, we will explore the utilization of deep learning technology for emotion recognition, developing a deep neural network structure suitable for emotional EEG signals and improving the classification accuracy. One possible solution to deal with this problem is to adopt stochastic configuration networks techniques [49].

## Figures and Tables

**Table 1:** Brief description of movie clips used in the emotion experiment.

No.	Label	Movie Name	Length (s)
1	Joy	More Haste Less Speed	109
2	Joy	Bie Na Zi Ji Bu Dang Gan Bu	142
3	Joy	Flirting Scholar	112
4	Neutral	IP Package	70
5	Neutral	Hardware Conflict	65
6	Neutral	IDE Interface Repair	77
7	Sad	My Brothers and Sisters	146
8	Sad	Mom Love Me Once More	136
9	Sad	Warm Spring	101
10	Disgust	Black Sun 731(1)	100
11	Disgust	Black Sun 731(3)	68
12	Disgust	Vomit	90
13	Anger	Fist of Fury (2)	66
14	Anger	Kangxi Dynasty	94
15	Anger	Conman in Tokyo	107
16	Fear	Help Me	50
17	Fear	The Game of Killing (1)	159
18	Fear	Inner Senses	86

**Table 2:** Parameter settings of EEG recording system.

Parameters	Settings
Amplifier	gtec.HIamp
Sampling Frequency	512 Hz
Band Pass Filter Frequency	0.1–100 Hz
Notch frequency	50 Hz
Electrode Layout	International 10–20 System
GND Electrode Position	AFz
Reference Electrode Position	Fz, Right Earlobe
Electrode Material	Ag/AgCl
EEG Recording Software	g.Recorder

**Table 3:** Frequency band ranges of EEG signals.

Frequency Band	Bandwidth (Hz)
*δ* (Delta)	1–4 Hz
*θ* (Theta)	4–8 Hz
*α* (Alpha)	8–12 Hz
*β* (Beta)	12–30 Hz
*γ* (Gamma)	30–64 Hz

**Table 4:** Accuracies of binary classification for the discrimination of positive emotions from negative emotions (Standard deviations are shown in parentheses).

No. Participant	Accuracy (%)		
	Movie-Induced Emotion Recognition	Self-Induced Emotion Recognition	Prediction of Self-Induced Emotion through Movie-Induced Emotion
1	93.33	87.48	82.99
2	99.84	98.91	87.21
3	97.01	99.73	95.51
4	90.20	88.71	86.94
5	94.01	82.59	93.61
6	97.41	85.17	86.12
7	93.47	76.46	78.91
8	99.86	99.32	93.06
9	86.94	82.04	73.33
10	74.69	96.05	80.14
11	89.93	87.62	80.95
12	87.48	92.65	81.63
13	89.52	77.14	48.84
14	67.89	68.16	69.93
15	92.65	90.20	88.98
16	82.04	65.58	50.07
17	77.69	94.69	56.05
18	86.26	79.05	88.44
19	71.70	82.72	96.46
20	87.07	90.07	79.32
21	69.52	85.17	81.90
22	79.18	85.71	31.02
23	93.20	87.35	48.57
24	87.21	90.88	82.85
25	86.80	87.35	60.14
26	97.96	97.82	99.32
27	88.98	86.12	80.82
28	89.93	92.80	89.93
29	86.53	91.43	98.78
30	77.69	91.84	84.08
Average	87.20 (8.74)	87.36 (8.19)	78.53 (16.66)

**Table 5:** Performance of binary classification for the discrimination of positive emotions from negative emotions (Standard deviations are shown in parentheses).

		Movie-Induced Emotion Recognition		Self-Induced Emotion Recognition		Prediction of Self-Induced Emotion through Movie-Induced Emotion	
	Label	Positive	Negative	Positive	Negative	Positive	Negative
Predict							
Positive		2788	1200	2667	1044	2578	2902
Negative		1622	16440	1743	16596	1832	14738
Positive F1-Score		0.66		0.67		0.52	
Negative F1-Score		0.94		0.92		0.86	
Accuracy (%)		87.20 (8.74)		87.36 (8.19)		78.53 (16.66)	

**Table 6:** Accuracies for the classification of emotions into six discrete categories. (Standard deviations are shown in parentheses).

No. Participant	Accuracy (%)		
	Movie-Induced Emotion Recognition	Self-Induced Emotion Recognition	Prediction of Self-Induced Emotion through Movie-Induced Emotion
1	65.65	63.04	65.65
2	65.87	56.24	47.28
3	56.01	63.72	50.00
4	39.68	57.71	66.55
5	51.93	50.45	69.05
6	55.10	53.74	42.06
7	57.03	40.82	33.79
8	72.45	71.77	47.51
9	41.27	64.29	40.93
10	52.39	39.57	54.20
11	56.92	52.95	39.68
12	60.32	54.20	46.94
13	39.46	46.15	40.36
14	41.38	58.05	64.51
15	54.42	56.69	41.27
16	43.65	40.82	41.95
17	42.52	60.66	46.71
18	65.99	44.67	51.25
19	49.89	64.29	56.92
20	60.20	55.33	31.07
21	73.24	70.52	29.48
22	41.27	41.16	42.74
23	60.54	29.59	48.64
24	58.50	58.50	60.54
25	51.70	47.05	26.76
26	81.52	82.54	85.94
27	62.13	64.63	51.47
28	55.22	49.43	48.30
29	59.52	44.78	69.16
30	53.63	52.38	56.92
Average	55.65 (10.39)	54.52 (11.02)	49.92 (13.09)

**Table 7:** Top 20 electrodes for the classification of emotions into six discrete categories (Electrodes are selected in accordance with MRMR ranking).

Type of Emotion	Features		
	Beta (DE)	Gamma (DE)	EMD (Dt)
Movie-Induced Emotion	T8	AF7, AF8, FP1, FP2, F6, F8, FC6, FT8, T7, T8, TP8, TP9, TP10, C5, C6, CP6, P8, O1, O2, Oz	T7, T8, C6
Self-Induced Emotion	/	AF7, AF8, FP1, FC5, FC6, FT7, FT8, T7, T8, TP7, TP8, TP9, TP10, C5, C6, CP6, P8, O1, O2, Oz	T8, TP10, C6, FT8,
